# Detecting potential cooperative network for tourist attractions in a destination using search data

**DOI:** 10.1371/journal.pone.0298035

**Published:** 2024-02-07

**Authors:** Xuankai Ma, Fang Han, Tian Wang, Simin Fan, Lin Ma

**Affiliations:** 1 College of Geography and Remote Sensing Sciences, Xinjiang University, Urumqi, China; 2 State Key Laboratory of Desert and Oasis Ecology, Xinjiang Institute of Ecology and Geography, Chinese Academy of Sciences, Urumqi, China; 3 University of Chinese Academy of Sciences, Beijing, China; Rikkyo University, JAPAN

## Abstract

This study addresses the critical need for regional tourism integration and sustainable development by identifying cooperation opportunities among tourist attractions within a region. We introduce a novel methodology that combines association rule mining with complex network analysis and utilizes search index data as a dynamic and contemporary data source to reveal cooperative patterns among tourist attractions. Our approach delineates a potential cooperative network within the destination ecosystem, categorizing tourist attractions into three distinct communities: core, intermediary, and periphery. These communities correspond to high, medium, and low tourist demand scales, respectively. The study uncovers a self-organizing network structure, driven by congruences in internal tourist demand and variances in external tourist experiences. Functionally, there is a directed continuum of cooperation prospects among these communities. The core community, characterized by significant tourist demand, acts as a catalyst, boosting demand for other attractions. The intermediary community, central in the network, links the core and periphery, enhancing cooperative ties and influence. Peripheral attractions, representing latent growth areas within the destination matrix, benefit from associations with the core and intermediary communities. Our findings provide vital insights into the dynamics, systemic characteristics, and fundamental mechanisms of potential cooperation networks among tourist attractions. They enable tourism management organizations to employ our analytical framework for real-time monitoring of tourism demand and flow trends. Additionally, the study guides the macro-control of tourism flows based on the tourism network, thereby improving the tourist experience and promoting coordinated development among inter-regional tourist attractions.

## Introduction

The proliferation of tourism information coupled with the dynamic circulation of tourism data has underscored the increasing diversity and complexity of tourism demands [1]. In light of regional tourism integration, there is a pressing need to recalibrate tourism development strategies [2]. Set against this dual context, tourism routes among tourist attractions are poised for ongoing development and enhancement. This necessitates that tourist attractions within a region forge ahead with novel collaborations and synergies to rise to the challenge of the ever-evolving tourist market, and promote diversification of attractions experiences [3, 4]. At present, tourists stand to gain from the rampant competition in the tourism sector, endowed with an array of choices in tourism products and services, and a plethora of fallback options [5]. The organizational nexus of tourist attractions emerges as a byproduct of the symbiotic evolution among tourism spatial constituents [6]. This paradigm has been morphed from bilateral or hierarchical top-down to novel collaborations partnerships [7]. The spatial proximity of tourist attractions influences their competition and cooperation mechanisms, following the distance decay law [8]. However, in the space of network, attractions can maintain connections with distant attractions, expanding their influence beyond classic spatial limitations. Network science is widely used to describe interactions between spatial elements, allowing researchers to analyze the topological structural properties of tourism networks [9–11], which’s generation is amenable to control, and its intrinsic structure is susceptible to planning [12]. It is pivotal to recognize that while tourist attractions can gravitate towards self-organized networks with the maturation of the tourism sector, they fall short of achieving the zenith of cooperative network configurations [13].

The construction of networked tourist attractions significantly enhances the tourist experience by improving the environment, display, and interaction dimensions, thereby enriching cultural aspects of tourism destinations [14]. In the context of the European Union, cross-border tourism is bolstered by policies such as the Schengen Agreement, INTERREG Community Initiative, and Euroregions, which collectively facilitate destination development and sustainable tourism practices [15, 16]. These initiatives underscore the importance of innovation and knowledge transfer in regional development, emphasizing mobility, connectivity, and governance [17]. Similarly in China, the construction of high-speed railway networks has been identified as a key driver for tourism development, offering enhanced accessibility and connectivity to various tourist destinations. A recent study highlighted the positive coupling coordination relationship between tourism carrying capacity and the high-speed railway network, emphasizing the improved marginal effect on tourism industry development with the expansion of these networks [18]. These policy background and study cases are providing a strategic alignment support of network development of tourism attractions across regions with tourism promotion.

Internet search engines, through their provision of search indices, furnish regional tourism management organizations with a reflexive mechanism for the quantitative surveillance of evolving tourist intentions and preferences [19]. Such indices are demonstrably capable of capturing the nuanced oscillations in prospective tourism interest emanating from source regions towards destinations, inclusive of specific attractions, within distinct time frames [20]. This, in turn, paves the way for a deeper understanding of the latent collaborative potential and intricate networking dynamics among tourist attractions, viewed predominantly from a demand-centric perspective.

Consequently, there is an imperative to steer and modulate the genesis of the cooperative network, ensuring its optimal efficacy [21]. The crux of this research endeavor is to unearth a methodology that glimpses into the cooperative prospects among tourist attractions, subsequently modeling the regional tourism cooperative network. This endeavor will shed light on latent collaborative avenues between tourist attractions, and the empirical assessment of network functionality and efficiency will lay the groundwork for strategic network optimization.

## Literature review

### Internal collaborative features within a destination

In the context of destination management, the term ’destination’ encompasses not only a geographically bounded region but also a dynamic and multifaceted entity influenced by tourists’ subjective interpretations [22]. As Gunn (2013) aptly notes, a destination system comprises a network of loosely affiliated tourist attractions, akin to cooperative entities [23]. These attractions collaborate to offer visitors a more integrated and enriched travel experience, a concept commonly referred to as regional tourism integration [24]. Thrift’s (1996) perspective underscores that destinations are intricate environments where internal elements interact and evolve [25].

Collaborative interactions among various destination elements exhibit multi-agent (regions-communities, organizations-enterprises, institutions-administrations), multi-tier (individual, groups, organizations), and dynamic characteristics, which typically unfold within public spaces centered around tourism attractions [26]. However, several challenges, such as the tragedy of the commons, the prisoner’s dilemma, and collective action dilemmas, afflict the interactions between stakeholders in destination tourism and their associated institutions [27]. The tragedy of the commons arises due to the overexploitation of public tourism resources and the absence of unified pricing management [28]. The prisoner’s dilemma emerges because there is limited interaction among stakeholders concerning the information within the vertical industry, preventing actors from identifying cooperation opportunities [29]. Furthermore, the relationship between stakeholders and regional tourism management organizations forms a loose collective stemming from the tourism industry. The lack of a macro-level understanding of regional tourism demand hinders effective collective action [30]. Given that tourism attractions serve as the foundation and spatial conduits for collaborative endeavors among stakeholders, this study posits that investigating the relationships and networks among tourism attractions is paramount for uncovering collaborative opportunities and potential. This approach empowers regional tourism management organizations to foster formal cooperation based on effective information, thereby enhancing the operational efficiency of destination tourism.

The realm of tourism management has undeniably played a pivotal role in orchestrating and championing a "top-down" collaborative paradigm. Yet, this supply-centric approach, while well-intentioned, has occasionally been marred by its inherent rigidity, inadvertently promoting local protectionism and fostering an uneven competitive landscape [31]. Such critiques often underscore a perceived disconnect between this overarching strategy and the nuanced demands of the primary tourist market. By weaving in demand-side perspectives into the collaborative matrix of tourist attractions, there lies a promising avenue to amplify the effectiveness and resonance of tourism management initiatives. Tourists, driven by their individual social backgrounds, travel objectives, exposure to tourism-related information, educational backgrounds, and consumption patterns, contribute to the nuanced and subjective nature of destinations. Consequently, destinations are not solely defined by geographical boundaries but also by the diverse lenses through which tourists perceive them. Kalantari (2020) highlights that tourists’ inherent travel intentions shape their selection of specific tourist attractions within a destination, thereby influencing the quality of their travel experiences [32]. In this study, we maintain the conventional geographical definition of a destination while acknowledging the uniqueness of tourists’ perceptions. This dual perspective empowers destination management organizations to gain valuable insights into the intricate web of relationships among tourist attractions within destinations. These insights, originating from the demand side (tourist origins), play a pivotal role in facilitating regional tourism planning, marketing strategies, and initiatives such as collaborative branding and curated tour itineraries among tourist attractions [33].

### Tourist attractions network administration paradigm

The adoption of a network perspective offers destinations an innovative and efficient management framework [2], facilitating superior coordination and optimization of internal resources, thereby enhancing competitive positioning. Traditional public management of destinations is evolving towards a more localized approach, driven by the nuances of tourist demand [34–36]. This evolution suggests a recalibration in governance, transitioning from hands-on management to a role centered around fostering synergy among diverse tourist attractions within a destination [35, 37]. Such a transition underscores the significance of harmonizing inter-attraction relationships to carve out a competitive edge [38]. Furthermore, integrating a network-centric management approach not only streamlines coordination among internal attractions but also equips destinations to adeptly navigate external shifts [39], be it evolving tourist preferences, emerging tourism trends, or challenges stemming from tourism-related crises [33, 40]. This perspective accentuates the symbiotic relationships binding various tourist attractions. To deliver a holistic tourism experience, it’s imperative for these attractions to collaborate seamlessly, ensuring every facet of the destination resonates with the tourist’s expectations [41, 42]. When destinations are marked by a collaborative ethos among attractions, the collective benefits are manifold [43, 44]. Beyond immediate advantages, such a collaborative framework augments a destination’s resilience and propensity for innovation [41, 45], cementing its competitive stature in the ever-dynamic tourism landscape.

Research has illustrated how tourist attractions, as perceived in the minds of tourists, can be mapped onto a tourism network, uncovering both singular and multifaceted core-periphery structure [46]. This methodology challenges traditional notions that rely heavily on spatial distances when conceptualizing network structures of tourist attractions. The imperative now lies in understanding how these networks, built on tourists’ predilections, can influence the trajectory of tourist attraction development. Furthermore, the intricate spatial configurations stemming from potential symbiotic relationships between attractions warrant further investigation. There is a discernible trend in the adoption of distinct cooperation paradigms, both temporally and geographically. From a temporal perspective, certain attractions gravitate towards concurrent collaborations, aiming for a synergistic promotional effort, whereas others strategically stagger their cooperative endeavors to sidestep head-on competition during the zenith of the tourist season [47]. On the geographical front, a subset of attractions form alliances with proximate entities, curating a seamless tourist journey [48], while a contrasting group ventures into inter-regional collaborations, aiming to furnish tourists with an enriched tapestry of experiences [49]. Such nuanced cooperation modalities not only amplify the allure of the destination but also adeptly address the multifaceted desires of the modern tourist.

While a plethora of studies have highlighted the merits of utilizing a network perspective to elucidate the interconnections among tourist attractions, there is a conspicuous absence of research delving into the collaboration between these attractions from the lens of demand-side tourist preferences. More pointedly, scant attention has been paid to potential collaboration avenues steered by tourism demand. Against this backdrop, this study is poised to unravel two salient questions: How can data mining techniques be leveraged to map out potential cooperative network for tourist attractions within a destination, anchored in a demand-side viewpoint? What are the intrinsic structural nuances and cooperative types that underpin this network? By addressing these pivotal concerns, this research aims to augment our comprehension of demand-driven tourism cooperation networks and proffer both analytical instruments and theoretical touchstones that can galvanize holistic regional tourism management.

## Materials and methodologies

### Framework

**Fig 1:**
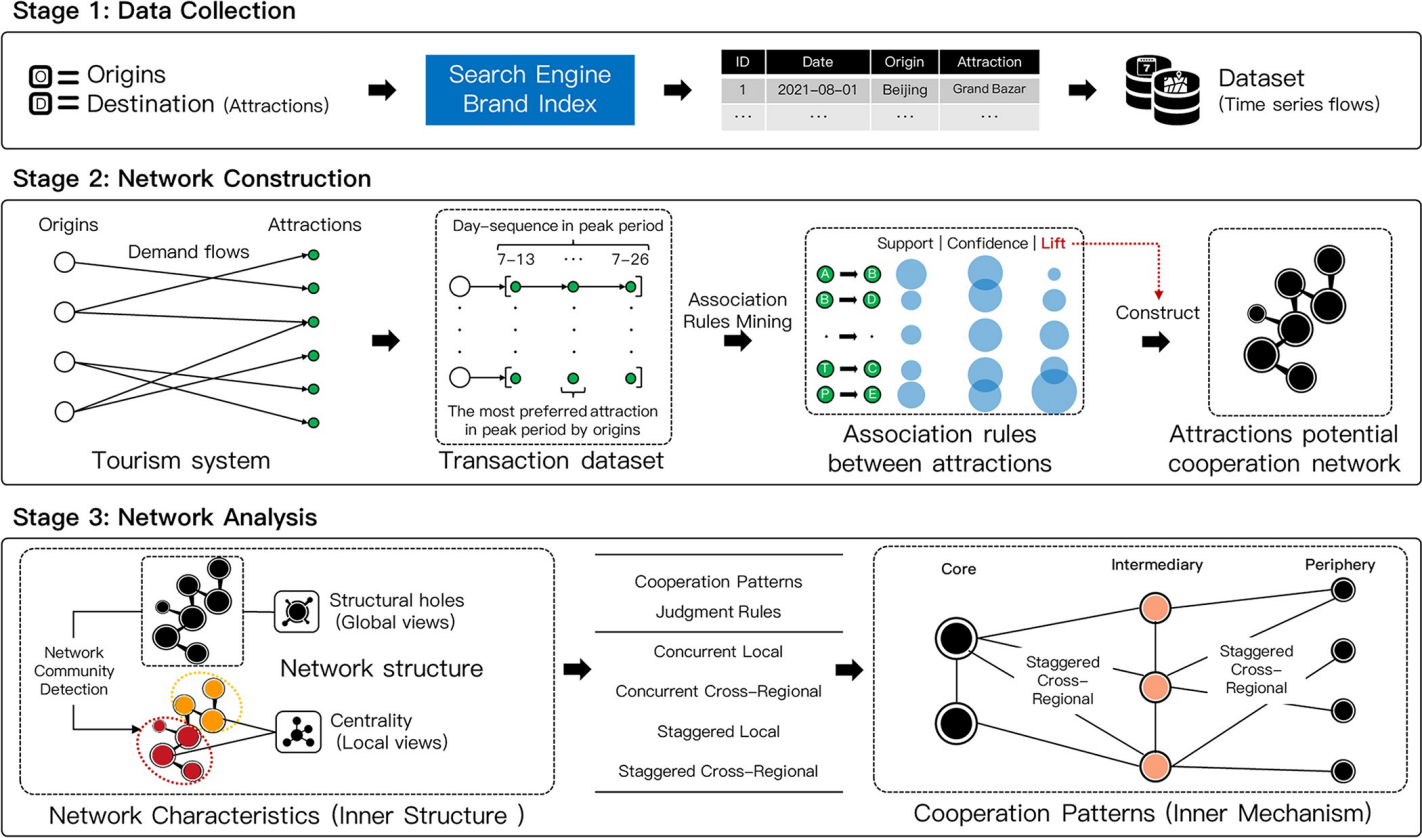
The research framework.

This research endeavors to devise a methodology that harnesses search indices to uncover potential collaboration opportunities among tourist attractions within a destination, all from a demand-side perspective. By modeling the collaborative network of attractions, we aim to delve into the network’s functionality and intrinsic structural attributes. This approach seeks to bridge existing research gaps and align with contemporary trends in the study of tourism collaborative networks (Fig 1).

For the empirical component of our study, we perceive China as an intricate tourism ecosystem. This system encompasses provinces that serve as origins, funneling tourists towards a regional destination teeming with diverse attractions. To emulate the dynamics of this system, we employ the innovative Baidu search brand index, which offers a continuous daily stream of data on origins-destination preferences, marking a departure from the conventional keyword index. We envision the destination as a metaphorical supermarket, stocked with a plethora of tourist attractions, each analogous to a unique product. Distinct origins symbolize different customer segments, and their purchasing behaviors reveal insightful association patterns. Mining these patterns can spotlight potential synergies between attractions. For instance, if a significant proportion of origins showing a preference for attraction A also exhibit a keen interest in attraction B, it hints at a latent affiliation between the two. Such revelations can be instrumental for tourism authorities when strategizing brand collaborations for attractions and fostering inter-regional partnerships.

By capturing the most preferred tourist attractions from origins during peak periods, we create a transaction dataset to mine association rules and construct a network among the tourist attractions. Moreover, community detection is crucial for decomposing the network and enables us to identify dominant attractions, subsequent attractions, mediated attractions within local communities, and isolated attractions that lack connections to other attractions. Assessing the relationships among tourism attractions based on judgment rules provides valuable insights into the intrinsic links within the network structure.

### Study case

**Fig 2:**
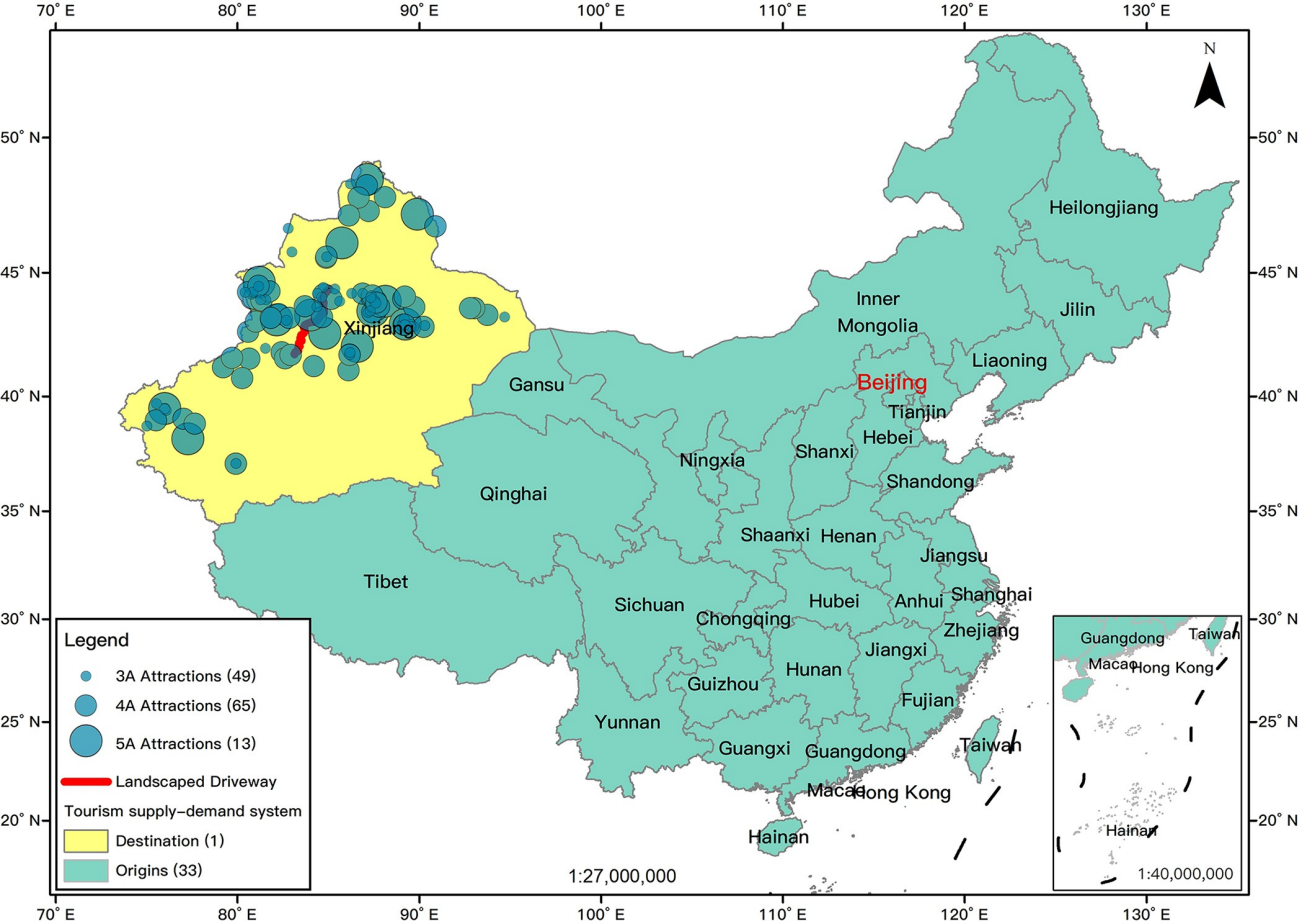
The study case. The standard map services are provided by the Ministry of Natural Resources of China (http://bzdt.ch.mnr.gov.cn/), GS (2021)5448.

Xinjiang, located on the north-western border of the People’s Republic of China, is the largest provincial administrative unit in China in terms of area (Fig 2). As the most active area of the ancient silk road, Xinjiang’s unique natural and cultural environment created a prosperous and rare natural landscape, a splendid ancient civilization, and varied ethnic customs. Furthermore, excellent tourism demand emerged for tourists in the east of China to visit Xinjiang caused of the heterogeneity of the landscape and culture and the distance of Xinjiang. In June 2022, Xinjiang received 23.92 million tourists and achieved a tourism revenue of 17.412 billion yuan. From July 1 to July 8, 2022, the average daily reception of 5A tourist attractions exceeded 110,000 person-visits, with the actual number of ticket buyers in Nalati, Kanas, Tianchi, Sayram Lake, and Cocotohai above 10,000 per day. Thus, Xinjiang and the Chinese domestic tourism system were representative cases for investigating the potential cooperative network for tourist attractions.

### Dataset

Previous studies have extensively employed the Search Index to measure tourism demand or tourist behaviors [19, 50–52]. An enhanced version, the Search Brand Index from Baidu, has emerged, surpassing the keyword-based approach. Unlike the Search Index, which relies on keyword queries, the Search Brand Index categorizes information based on standard industry divisions. Including comprehensive and informative industry and brand-related search terms enables a more comprehensive and accurate visual analysis, effectively portraying the evolving trends and demand characteristics of Internet users’ interests in industries, market segments, and brands.

**Fig 3:**
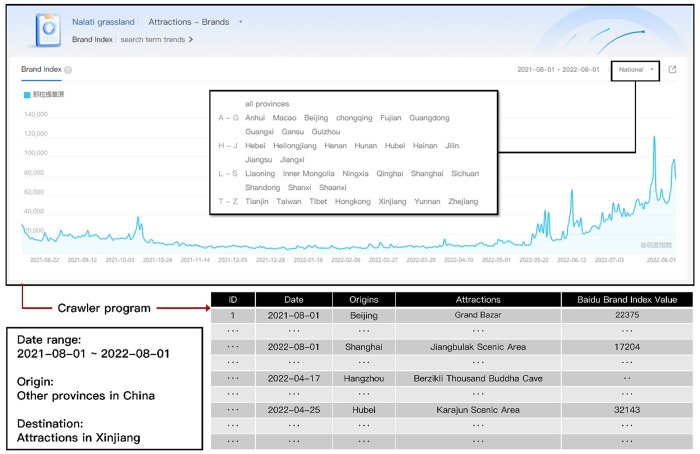
The Baidu Brand Index interface and sample data. The interface URL is https://index.baidu.com/v2/main/index.html#/brand/100639921?words=%E9%82%A3%E6%8B%89%E6%8F%90%E8%8D%89%E5%8E%9F.

To conduct our research, we leverage Baidu, the largest Chinese Internet search engine, as our primary data source. The Baidu Brand Index consolidates all relevant keywords associated with tourist attraction brands, allowing for calculating attraction demand across various channels and products. This comprehensive index, depicted in Fig 3, facilitates a more holistic understanding. Moreover, by leveraging users’ attributions, we can dynamically query the Baidu Brand Index, thereby constructing a dataset that captures the ebb and flow of tourist activity.

This paper employs the Baidu brand index as a reliable indicator for quantifying tourism demand for attractions. The data utilized in our analysis spans from August 1, 2021, to August 1, 2022, with a daily time resolution. We focus on attractions as the geographical unit of the destination while considering the provinces as the geographical unit for Internet users, who serve as the source of tourists. To acquire the necessary data, we selected 127 attractions rated 3A-5A and incorporated a landscaped driveway in Xinjiang as a crucial brand keyword parameter. Furthermore, we encompassed 33 other provinces in China to account for the regions to which the netizens belonged. Through this comprehensive approach, we gathered a total of 1,582,218 records (Table 1), capturing the continuous flow of tourism demand from August 1, 2021, to August 1, 2022 (Fig 3). Our analysis identifies a significant peak period for tourism demand, spanning from July 13 to July 26, 2022. This finding is supported by applying the Wavelet method [53, 54]. We performed association rules mining and network analysis based on this peak period to gain deeper insights into the data.

**Table 1:** Descriptive statistics of dataset.

Item	Obs.	Mean	Sd	Min	Max
**Attractions**	128	759,397	2,177,422	130	13,453,282
**Origin provinces**	33	2,945,539	2,472,535	13,986	9,586,130
**Tourism demand flows**	3,979	24,428	91,181	1	1,379,210

The value of each item is based on the summation of the study period.

### Methods

#### Association rule learning

Association rule learning is a machine learning technique designed to uncover relationships among items within large-scale datasets. This approach has found extensive applications in various domains, including tourism system recommendations [55], tourist preference analysis [56], and the study of spatiotemporal tourist behaviors [57]. In the present study, we commence by identifying the needs that arise from tourist source regions. To build on this initial step, we then turn our attention to tourist destinations that experience the highest daily demand during the peak period, specifically from July 13 to July 26, 2022, across 33 tourist origin provinces. These results are organized into a 33 x 12 matrix. A sample dataset is shown in Table 2. The Apriori algorithm is then deployed to extract association rules among these tourist attractions. These rules, once identified, act as the foundational elements for constructing a network of tourist attractions.

**Table 2:** The sample of the transaction dataset.

Origin	Sequence of attractions in tourism peak period				
	July 13	July 14	…	July 25	July 26
**Beijing**	White Sand Lake	Xinjiang Museum	…	Duku Road	Duku Road
**…**	…	…	…	…	…
**Shanghai**	White Sand Lake	Xinjiang Museum	…	Duku Road	Duku Road
**…**	…	…	…	…	…
**Guangdong**	Sayram Lake	Xinjiang Museum	…	Duku Road	Duku Road

The transaction dataset is generated from a matrix composed of tourist origins (33) multiplied by the highest search index attraction in each day in tourism peak period (12).

In association rule learning algorithms, Support, Lift, and Confidence serve as the three fundamental metrics for assessing the significance, correlation, and trustworthiness of association rules, respectively. Support quantifies the prevalence of an itemset within the entire dataset and is employed to eliminate infrequent itemset. Lift evaluates the independence between items A and B: a value greater than 1 signifies a positive correlation, less than 1 indicates a negative correlation, and equal to 1 suggests no correlation. Confidence measures the conditional probability of item B occurring when item A is present, serving as an indicator of the rule A→B’s reliability. Experiments were conducted with a minimum support of 0.05, a minimum confidence of 0.85, and a minimum lift of 1, yielding a total of 69 association rules.

#### Network analysis

Complex Network Analysis serves as a pivotal instrument for the graph-theoretical exploration of intricate social systems, and it has increasingly become a dominant framework for investigating the architecture of tourism networks [10, 58]. Within this context, the nonparametric Bayesian inference offers a distinct advantage over conventional community detection algorithms such as the Louvain algorithm, spectral clustering, and hierarchical clustering. Specifically, it eliminates the need for a priori specification of community numbers and obviates the requirement for manual cross-validation. This unique feature allows the methodology to autonomously adapt to the nuanced complexities of the network, thereby excelling in the identification of a statistically significant array of heterogeneous communities within tourism networks. The modularity Q is a metric of community detection, which is in range of typically falls between -1 and 1. A Q value approaching 1 signifies a strong community structure within the network, while a value near 0 indicates a lack of distinct community organization. Values close to -1 are uncommon and suggest that the network’s community structure deviates from expectations. To further analyze the tourism network, Complex Network Analysis tools are employed. Centrality metrics are utilized to ascertain the functional roles and significance of individual nodes within these communities. Concurrently, structural holes analysis provides insights into gaps in connectivity across the global network, thereby complementing the community detection efforts. In addition, the above analysis tools and algorithms are all supported by Gephi software.

#### Spatiotemporal classifications for cooperation patterns

In the present study, a novel methodology is put forward to delineate collaboration patterns observed among tourist destinations (Table 3). Drawing on spatial and temporal dimensions, these patterns are systematically classified into four distinct categories: concurrent local, concurrent cross-regional, staggered local, and staggered cross-regional collaborations. From a spatial perspective, the categorization critically assesses whether tourist attractions fall within the same administrative boundaries. On the temporal front, the classification is underpinned by the Pearson correlation coefficients coupled with Granger causality test to process tourist attractions time series data, with a particular focus on both its immediate and lagged correlations.

**Table 3:** The classification for cooperation patterns between tourism attractions.

Patterns	Pearson Correlation	Lagged Correlation	Geographical Proximity
**Concurrent Local**	+(***)	Not significant	Same Region
**Concurrent Cross-Regional**	+(***)	Not significant	Different Region
**Staggered Local**	Not significant	+(***)	Same Region
**Staggered Cross-Regional**	Not significant	+(***)	Different Region

+/- denote positive or negative correlation

*** indicates statistical significance at a 0.01 level.

## Empirical results

### Association rules learning and network construction

#### Xinjiang attractions and association rules

**Fig 4:**
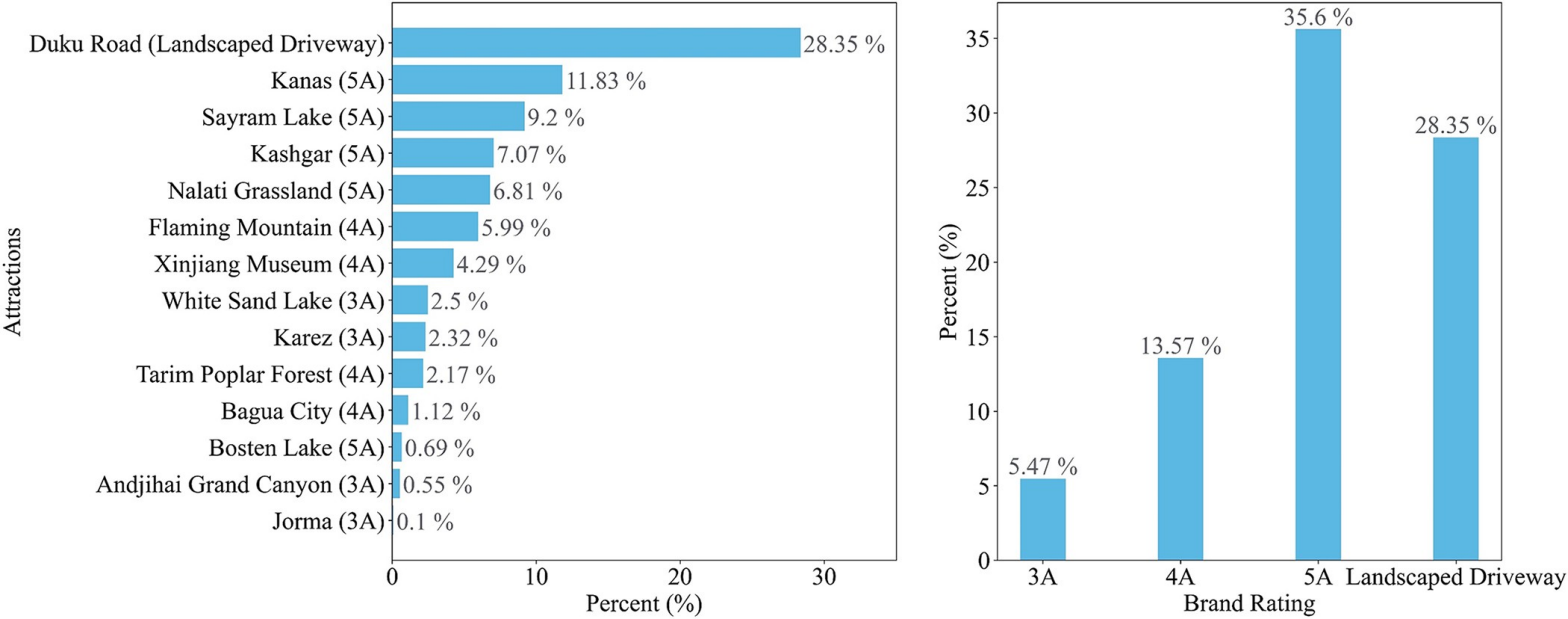
Proportion of attractions and brand ratings from transaction dataset.

After merging and de-duplicating the sequence of attractions, the 14 attractions significantly concerned by the origins, the tourism demand of which accounts for 82.99% of the total tourism demand during the peak period, are obtained. As shown in Fig 4, Duku Road, Kanas, and Sayram Lake are the most attractive brands. From the perspective of brand rating, the brand rating of attractions positively correlates with their tourism demand. The higher the rating, the more vigorous the tourism demand. It is worth mentioning that landscaped driveway is not currently included in the official tourism rating system, but landscaped driveway with elegant and heterogeneous natural landscape also has tremendous potential tourism demand and attractiveness.

Table 4 shows the top 15 association rules with the highest support, each consisting of antecedent and consequent, i.e., tourists like the antecedent (one or one or multi-attraction) while preferring the consequent (one or multi-attraction). When considering that antecedent tourist attraction exerts a positive influence on the tourism demand for consequent tourist attraction, it can be inferred that tourists express a desire for the potential synergy between these attractions, indicating potential collaboration opportunities. The Apriori algorithm’s elucidation, encapsulated as [X⇒Y support, rule support, confidence, lift. The antecedent and consequent, seamlessly bridged by ⇒, epitomize the conditions and ramifications of a potential cooperations among attractions. The triad of metrics—rule support, confidence, and lift—furnishes an exhaustive perspective of the association rules. The support degree is utilized to assess the accuracy of the association rules, which is indicated by the proportion of antecedents and consequences in the transaction data set. Confidence is a measurement of the probability of a consequence occurring in the presence of an antecedent. The lift indicator measures the occurrence of the antecedent and varies the probability of the occurrence of the consequence. When the degree of lift is >1, the antecedent has a lifting effect on the consequence.

**Table 4:** The top 15 association rules from frequent itemsets are sorted by support.

Antecedents (support)		Consequents (support)	Rules (support)	Confidence	Lift
White Sand Lake∧Duku Road 0.64	⇒	Flaming Mountain 0.88	0.64	1	1.14
White Sand Lake 0.64	⇒	Duku Road∧Xinjiang Museum 0.88	0.64	1	1.14
White Sand Lake 0.64	⇒	Flaming Mountain 0.88	0.64	1	1.14
Karez∧Duku Road 0.52	⇒	Flaming Mountain 0.88	0.52	1	1.14
Karez 0.52	⇒	Flaming Mountain 0.88	0.52	1	1.14
Karez 0.52	⇒	Flaming Mountain 0.88	0.52	1	1.14
Karez∧Duku Road 0.52	⇒	Flaming Mountain 0.64	0.48	0.94	1.48
Karez∧Xinjiang Museum 0.52	⇒	Flaming Mountain 0.64	0.48	0.94	1.48
White Sand Lake∧Karez 0.48	⇒	Flaming Mountain 0.88	0.48	1	1.14
Karez 0.52	⇒	Flaming Mountain 0.64	0.48	0.94	1.48
Karez 0.52	⇒	Flaming Mountain 0.64	0.48	0.94	1.48
Karez∧Xinjiang Museum 0.52	⇒	Flaming Mountain 0.64	0.48	0.94	1.48
Karez∧Duku Road 0.52	⇒	Flaming Mountain 0.64	0.48	0.94	1.48
White Sand Lake∧Karez 0.48	⇒	Duku Road∧Xinjiang Museum 0.88	0.48	1	1.14
Xinjiang Museum∧Karez∧Duku Road 0.52	⇒	Flaming Mountain 0.64	0.48	0.94	1.48

**Fig 5:**
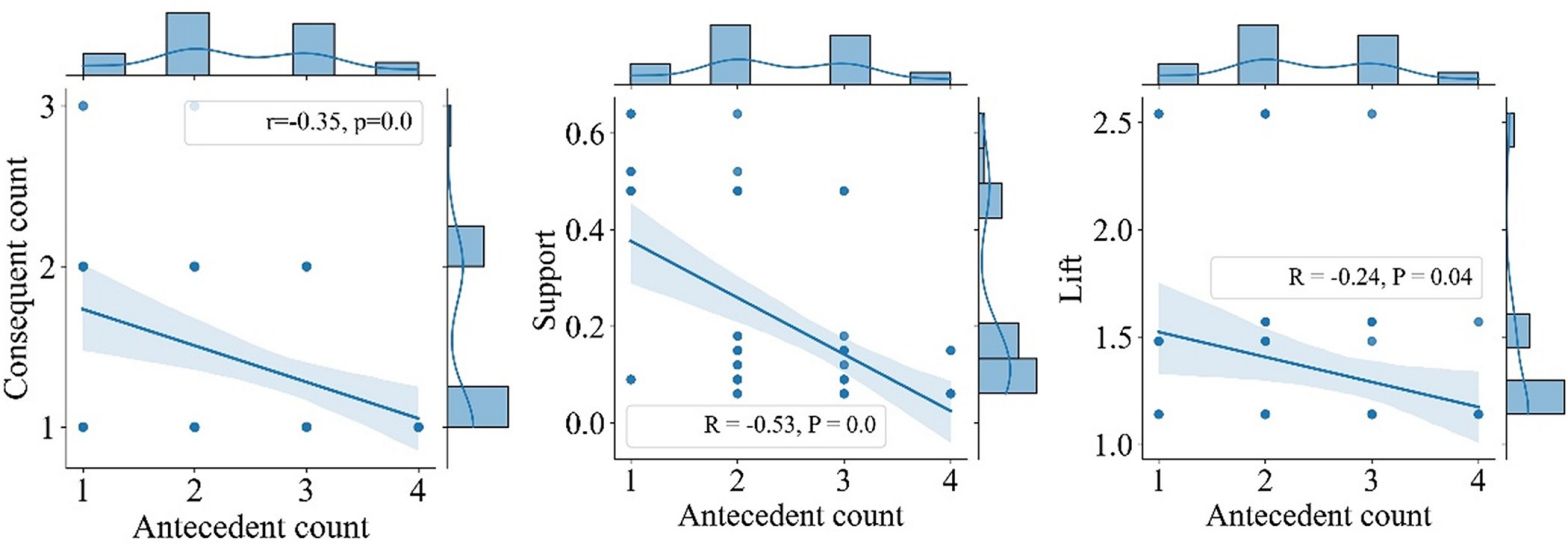
The Bivariate joint distribution diagrams of association rules characteristic based on linear regression.

Statistical analysis of the indicators of association rules reveals (Fig 5) that the number of antecedent tourist attractions in association rules is mainly two to three, and the number of consequence tourist attractions is primarily single. It shows a negative correlation, i.e., the more antecedent tourist attractions, the fewer the consequence tourist attractions. Moreover, with the increase in the number of antecedent tourist attractions, the support of the rule shows a significant decreasing trend. The enhancement effect of the antecedent tourist attractions on the consequent tourist attractions is limited, and the less the number of antecedent tourist attractions, the more evident the enhancement degree is. The findings above demonstrate that tourists in Xinjiang prefer cross-regional travel patterns rather than a comprehensive tour in a particular region. In addition, as the tourism resources are highly heterogeneous among tourist attractions in Xinjiang, cooperation, and complementarity among cross-regional attractions are the main modes of symbiotic mechanisms for tourist attractions in the region.

#### Generating potential cooperative network for tourist attractions

**Fig 6:**
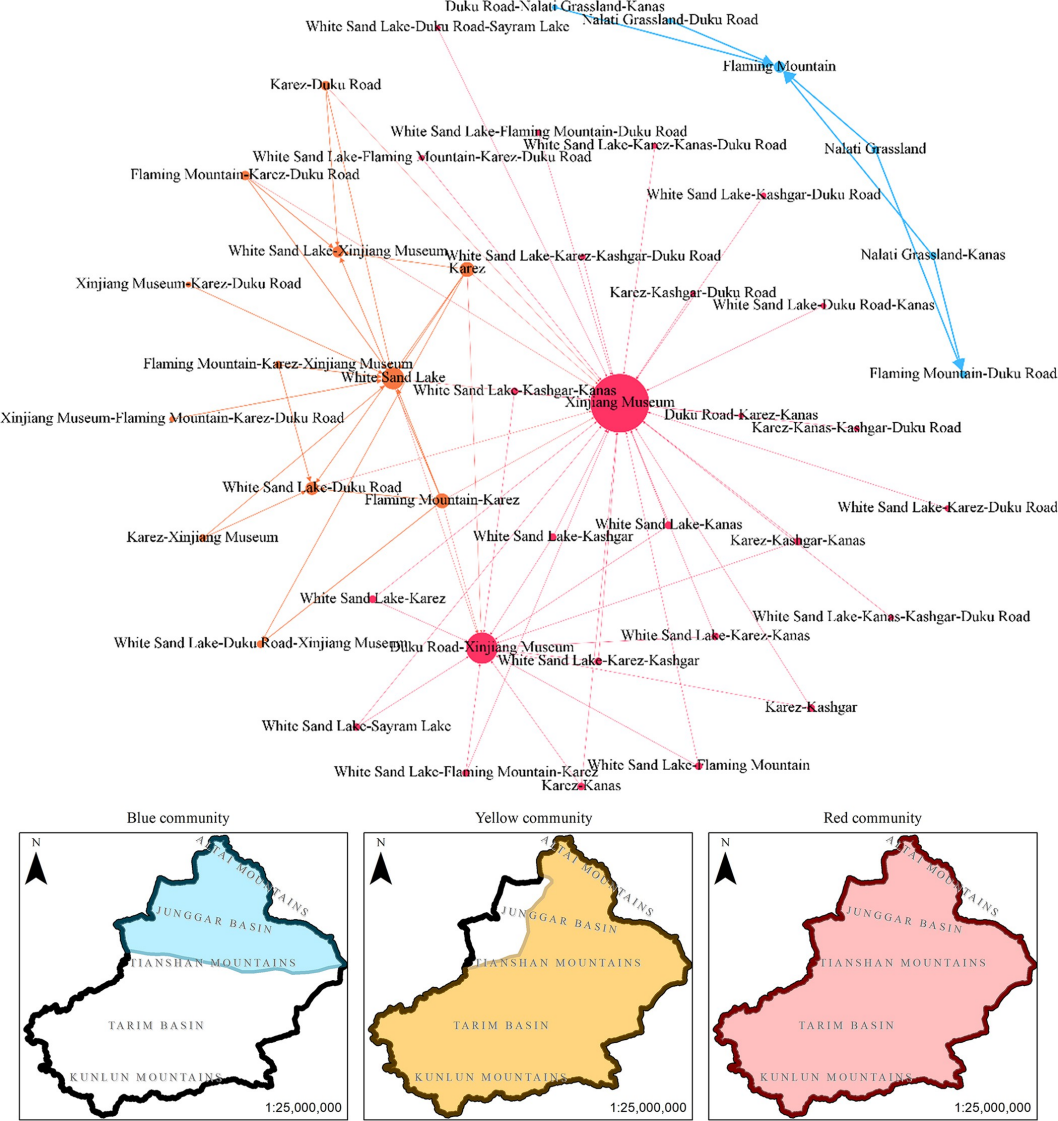
The structures of tourism communities in the potential cooperative network for tourist attractions.

Sixty-nine association rules are mapped into a network to identify the association relationships between attractions by probing the network’s structural characteristics for this investigation. The detailed procedure is as follows: the antecedents and consequences in the association rules are transformed as nodes in the network. The logical relationship between the antecedents and consequences is a directed edge between two nodes. The lift indices of the association rule weigh the directed edges. Notably, the two nodes which consist of the same attractions (e.g., Node {A, C}) and Node {C, A}) will be merged. Fig 6 shows the potential cooperative network for tourist attractions consisting of 44 nodes and 69 directed edges, which forms two independent (Non-connected) sub-networks.

### Network characteristics

#### Community detection

To delve deeper into the structure of potential cooperative network for tourist attractions in the study case, we employed the community detection algorithm. This approach shed light on clusters that have close association relationships within the network. Our empirical investigation pinpointed three distinct communities within this potential cooperation network, as corroborated by a modularity Q value of 0.503. Such a discovery underscores that cooperations between attractions are not merely random occurrences but follow a discernible pattern. The evident clustering might be attributed to overlapping tourism demands stemming from diverse tourist origins.

For visualization purposes, we color-coded the communities: blue, yellow, and red. The magnitude of the nodes was determined by their weighted degree in a broader context. As depicted in Fig 6, the variance among communities is manifested in the potency of the lifting effect. The blue community, resembling an isolated island (devoid of connections with other communities), stands out as the community with the most straightforward structure. It boasts an enhanced lifting degree among attractions and predominantly spans the northern region of Tianshan, carving out a triangle in Turpan-Altai-Ili. In contrast, the yellow community exhibits a more intricate network structure, with moderate lifting effects between attractions. It is geographically situated to the east of the Altai-Central Xinjiang Tianshan-Kashgar line. The red community, teeming with nodes and characterized by multiple attractions, sprawls across Xinjiang. However, the lifting effect between tourist attractions remains subdued.

#### Centrality

Centrality indicators serve as tools to elucidate the role of attractions within nodes in each community’s network structure. Specifically, in-degree centrality sheds light on the extent to which a node is lifted by other nodes. Conversely, out-degree centrality unveils a node’s capacity to amplify the tourism demand of other nodes. Betweenness centrality pinpoints nodes that assume an intermediary position in the network, exerting influence over other nodes. Closeness centrality is instrumental in singling out nodes that exhibit minimal dependence on other nodes.

**Fig 7:**
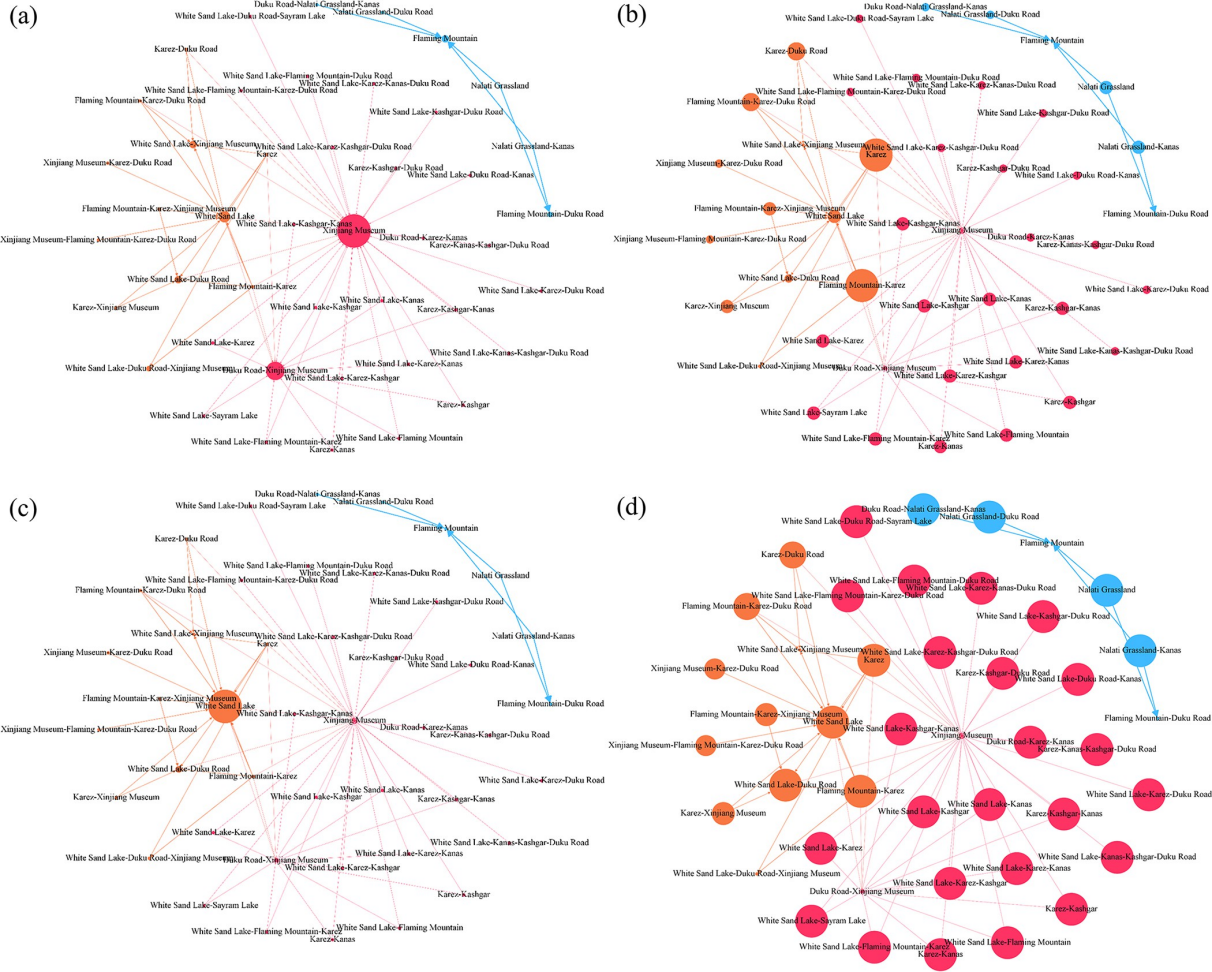
Attractions potential cooperation network structure based on node centralities. (a) In-degree centrality; (b) out-degree centrality; (c) betweenness centrality; (d) closeness centrality.

Fig 7 suggests that in-degree centrality and out-degree centrality develop a contrary and complementary formation, and this formation characteristic manifests that the lifting relationship between attractions tends to be unidirectional. More specifically, the Flaming Mountain in the blue community with high-level lifting is a typical lifted attraction. In contrast, the White Sand Lake in the yellow community with a mid-level of lifting is lifted by a combination of attractions within it and a few from other communities. The two outstanding attraction combinations in the red community with low-level lifting are the Xinjiang Museum and the Duku Road-Xinjiang Museum. In general, tourists from origins visiting the Nalati Grassland or the Nalati Grassland-Kanas lift tourism demand to the Flaming Mountains (within the blue community). Within the yellow community, the eastern region of Xinjiang, Karez, and Flaming Mountain-Karez have a lifting effect on tourism demand to White Sand Lake in the southern region of Xinjiang through the Duku Road (a scenic boulevard that traverses the north and south of the Tianshan Mountains) connection. Within the red community, the Xinjiang Museum and Duku Road-Xinjiang Museum are extensively lifted by other attractions. Although the single lifting effect is limited, the scale effect also makes both the advantageous beneficiaries of being lifted in the network.

White Sand Lake and White Sand Lake-Duku Road are the most significant attractions in the network, and their betweenness centrality indicates irreplaceability and plays a central intermediary role in the lifting network among attractions in Xinjiang. The network structure plotted by closeness centrality demonstrates that most attractions in Xinjiang have high liftable paths. Notably, Flaming Mountain, White Sand Lake-Xinjiang Museum, White Sand Lake-Duku Road-Xinjiang Museum, Xinjiang Museum, and Duku Road-Xinjiang Museum have very low closeness centrality attributed to their role in the network as being in a lifting relationship.

#### Structural holes

**Fig 8:**
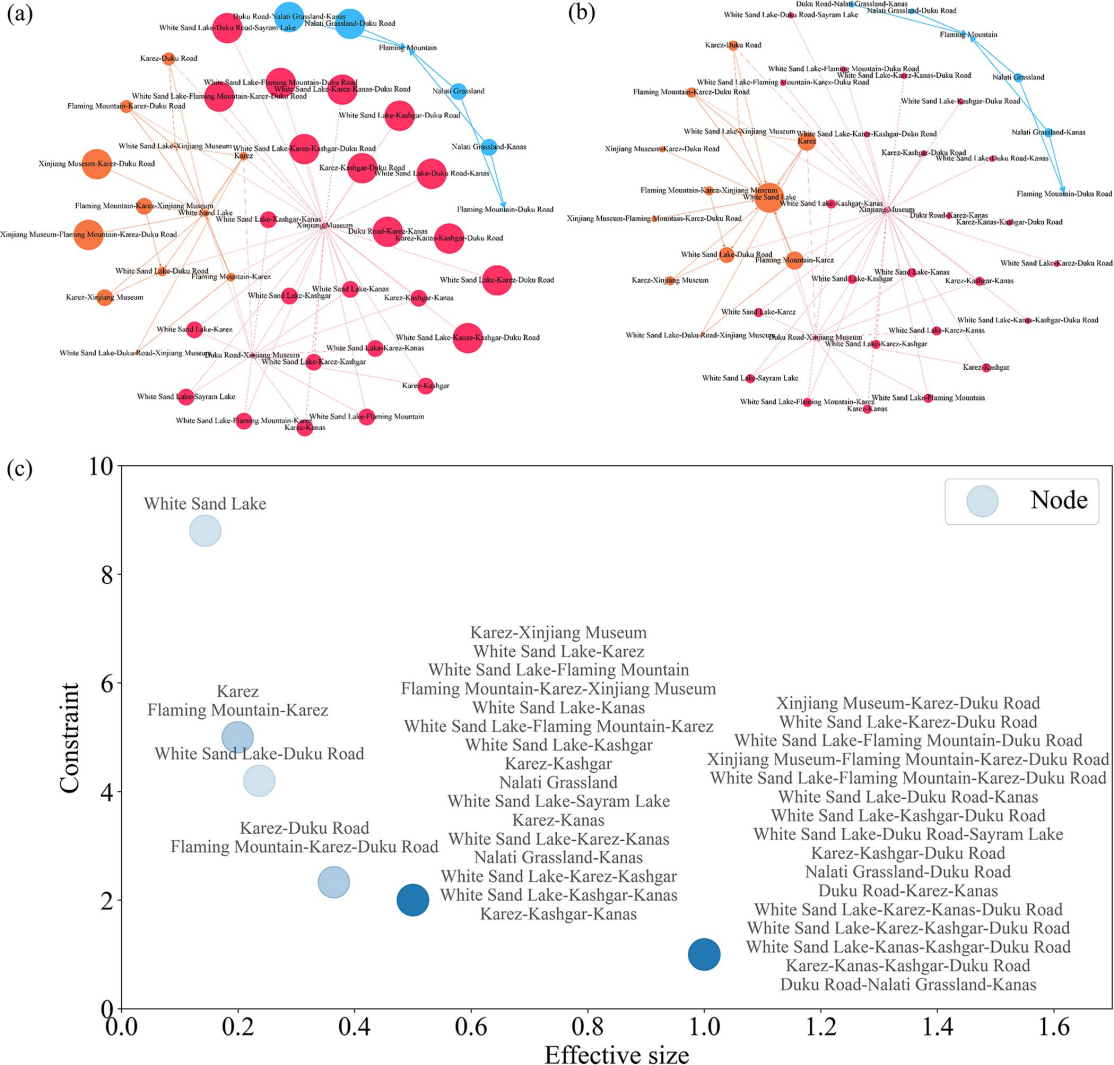
Attractions potential cooperation network structure based on structural holes. (a) Effective size; (b) constraint; (c) scatter plot of effective size and constraint.

Effective size and constraint are taken to evaluate the structural holes, which can show the position of the nodes in the potential cooperative network for tourist attractions and the strength of the relationship between these structures. The larger the effective size value, the more central the node is in the network. The smaller the constraint of the node, the less constrained the node is, indicating that these nodes act as a bridge between more nodes and are less likely to be controlled by others. By comparison with the results of the structural hole indicators, the effective sizes and the constraints are illustrated in a scatter plot (Fig 8) based on the network structure, which allows the roles of the nodes to be distinguished. The findings demonstrate that nodes formed by the combination of multiple attractions have a higher effective size and lower constraint degree, which enable them to have less constraint from other nodes in the network and have significant lifting dominance over other nodes to occupy the core position in the network. Contrariwise, White Sand Lake in southern Xinjiang, Karez, and Flaming Mountain in eastern Xinjiang have a low effective size and high constraint degree. Nevertheless, they are not significantly lifted by several other nodes in the network, owing to their high connectivity and intermediary role (Fig 7), which makes them constrained by other nodes.

In a comprehensive view, the left and right of the association chain within the attractions potential cooperation network in Xinjiang have robust and powerful lifting efficiency, while the middle part shows significant instability. It is expressed by transforming it into a lifting chain as follows: The Blue Community ({Nalati Grassland|Kanas}->*{Flaming Mountain}) = > Yellow Community ({Flaming Mountain*|*Kanas}->{White Sand Lake}) = > Red Community ({White Sand Lake*|Kashgar|Sayrem Lake}->Xinjiang Museum), where the italicized characters indicate the non-robust part of the chain and the structural hole of the attractions potential cooperation network. The attractions in the structural hole share the same features, i.e., a single type of tourism resource, spatially isolated and with limited heterogeneous-landscape attractions adjacently.

### Core-intermediary-periphery structure

Based on the analysis of network structure centrality and structure holes, the Xinjiang attractions potential cooperation network reveals a directed association chain. This indicates that tourists’ preferences contribute to the formation of an attractions potential cooperation network and establish directional relationships among attractions. The chain formation relies on the strong brand influence of Duku Road and the crucial role of transportation connections. To begin with, the left side of the chain is situated in the core tourism triangle of northern Xinjiang, with the Turpan Flaming Mountain as the primary attraction. As an antecedent attraction, Flaming Mountain and Karez stimulate tourism interest in White Sand Lake. This elevated interest in White Sand Lake, combined with Kashgar and Sayrem Lake, further boosts the tourism demand for the Xinjiang Museum, as observed on the right side of the chain. It is important to note that other attractions enhance the influence on subsequent attractions by forming connections with Duku Road. This road serves as a vital link among communities, enabling the formation of association chains with a loosely structured landscape driveway and three communities. The dominant attractions in the Blue Community possess significant lifting capabilities for homogeneous attractions but within a limited spatial scope. On the other hand, the sub-dominant attractions in the Yellow Community occupy a well-connected intermediary position within the network. They primarily act as transit stations, moderately influencing the attractions in the Red Community. Consequently, the destination system mostly benefits from the trickle-down effects and extensive lifting impacts generated by the dominant attractions.

The tourist attractions potential cooperation network within Xinjiang’s destination system comprises core, intermediary, and peripheral attractions, forming a core-intermediary-periphery structure. Table 5 provides the attributes of potential communities within this network. These communities’ centrality and structural hole attributes are determined by the average levels of their internal nodes. The blue community represents high-level attractions with the highest tourism demand. Despite having fewer nodes, it exhibits dense connections, making it the core or highly concentrated core community within the tourism attraction network. It serves as the primary driver for transferring and diffusing tourism demand throughout the network. The yellow community demonstrates moderate tourism demand intensity. With more nodes and higher connectivity, it acts as an intermediary, linking the core and peripheral communities. It expands the blue community’s enhancing role in the red community’s tourism demand. The red community features a smaller tourism demand scale. Lower-level attractions benefit from the enhancing effects of higher-level attractions through connections with intermediary attractions. Although it has more nodes, the connectivity within the community is lower, signifying its peripheral status with a somewhat diminished influence. Nonetheless, it contributes to maintaining the overall integrity and connectivity of the network.

**Table 5:** Table of community attributes within the tourism attraction network.

Community	Blue	Yellow	Red
**In degree**	1	1.5	1.731
**Out degree**	1	2.25	1.385
**Degree**	2	3.75	3.115
**Degree centrality**	0.047	0.087	0.072
**In-degree centrality**	0.023	0.035	0.04
**Out-degree centrality**	0.023	0.052	0.032
**Effective size**	0.5	0.376	0.692
**Closeness centrality**	0.667	0.678	0.923
**Constraint**	1	2.806	1.385
**Betweenness centrality**	0	0	0
**Node number**	6	12	26

The high degree of modularity in the tourist attractions potential cooperation network reveals that the core-intermediary-periphery structure plays a vital role in the evolution of regional tourism destinations. In well-established destination systems, the backbone of the tourist attractions potential cooperation network consists of high-level core attractions and main scenic routes. Meanwhile, lower-level peripheral attractions connect to the core attractions through intermediary attractions, actively contributing to the tourist attractions potential cooperation network. It highlights the significant connections between core attractions and the interplay between core, intermediary, and peripheral attractions during peak tourism periods in highly developed destinations. The core-intermediary-periphery structure facilitates the dissemination of tourism demand from high-level attractions to lower-level attractions, ultimately enhancing the overall tourism appeal of the destination.

#### Core

The core component comprises highly competitive tourism attractions with significant tourism demand. These attractions are widely recognized as prominent domestic or international tourist destinations, serving as the central hubs within the network and attracting a substantial flow of tourists and tourism resources. Core attractions are closely interconnected, forming a concentrated core area akin to an exclusive club. This interconnectedness among core attractions generates the "rich club effect." For instance, in the empirical findings, the Duku Road, Kanas, and Narat stand out as the most popular tourist attractions during peak periods and as a tightly connected community within the tourism attraction network. These three attractions account for 43.56% of the total tourism demand in Xinjiang. Their combined influence has the strongest impact on enhancing other tourist attractions (as indicated by the highest Lift value in the association rules).

#### Intermediary

The intermediary component is adjacent to the core attractions, where intermediary tourist attractions play a pivotal role as bridges between core attractions and peripheral attractions. They facilitate the flow of tourism demand and resources, enabling the exchange and dissemination of information between the core and peripheral attractions. Intermediary tourist attractions typically possess moderate tourism demand and competitiveness, connecting different regions and communities within the network. Their presence allows visitors to explore better and experience the diversity and richness of the destination, fostering overall network interconnectedness. For instance, the yellow community acts as the intermediary within the network, exhibiting mechanisms of unidirectional associations internally. The Flaming Mountains serve as a vital hub, connecting the blue community with the yellow community, while White Sand Lake acts as a significant gateway, linking the yellow community with the red community. Karez and the Duku Road play internal bridging roles within the yellow community. Notably, although the Duku Road is a core tourist attraction within the blue community, it connects with numerous other tourist attractions in the network, collaborating to enhance their impact. Many tourist attractions within the red (peripheral) community receive influences from the yellow (intermediary) community, which connects to core attractions but also benefits from connections with the Duku Road, receiving enhancing effects from other tourist attractions.

#### Periphery

Finally, the peripheral component comprises tourism attractions with relatively lower visitor traffic and less competitiveness. These attractions are situated in the outskirts or more remote areas of the destination, resulting in smaller scales of tourism demand. Nonetheless, peripheral tourist attractions still contribute to the overall connectivity and integrity of the destination. They introduce diversity and uniqueness, offering visitors distinct tourism options.

### The cooperation mechanism of network

To delve deeper into the potential collaboration patterns among tourist attractions within the study area, we integrated association rules with the potential cooperation network of tourist attractions. This process yielded four pivotal collaboration combinations (Table 6). These combinations mirror the core-intermediary-periphery structure within the network. Spatially, these combinations predominantly indicate a trend towards cross-regional collaboration, especially when intermediary attractions interact with other components. However, attractions within the intermediary category tend to collaborate more within the same region. These observations will serve as parameters for determining collaboration pattern classifications.

**Table 6:** The attractions from association rules and potential cooperation network.

Antecedents	Antecedents Community	Consequents	Consequents Community	In The Same Region
**Nalati Grassland**	Core	Flaming Mountain	Intermediary	FALSE
**White Sand Lake**	Intermediary	Flaming Mountain	Intermediary	FALSE
**Karez**	Intermediary	Flaming Mountain	Intermediary	TRUE
**White Sand Lake**	Intermediary	Xinjiang Museum	Periphery	FALSE

Table 7 meticulously delineates the cooperation patterns between various tourist attractions, substantiated by rigorous statistical measures. Firstly, focusing on the discerned cooperation patterns, both the “White Sand Lake-Xinjiang Museum” and “Nalati Grassland-Flaming Mountain” combinations are characterized by a “Staggered Cross-Regional” cooperation pattern. This pattern suggests a temporal disparity in their popularity or tourist influx, indicating potential benefits from temporally offset promotional endeavors. Such a pattern is particularly intriguing given that these attractions belong to different communities within the network, emphasizing the potential for cross-regional collaborations. The geographical proximity, or lack thereof, between these attractions further accentuates the significance of this staggered cooperation, as they are not situated in the same region. Secondly, the combinations “White Sand Lake-Flaming Mountain” and “Karez-Flaming Mountain” do not exhibit any significant cooperation pattern. The absence of a discernible pattern might be attributed to several factors. The Pearson P-values for these combinations surpass the conventional 0.01 threshold, suggesting that the observed correlations might not be statistically significant at a 99% confidence level. This lack of significance could arise from the inherent operational dynamics of these attractions, their positioning within the network, or external factors influencing tourist flows. Lastly, considering the community affiliations of the attractions within the network, it is noteworthy that the “Karez-Flaming Mountain” combination, despite both attractions being part of the intermediary community, does not manifest a significant cooperation pattern. This observation is intriguing, especially given their geographical proximity, as they are located in the same region. Such anomalies underscore the multifaceted nature of tourist preferences, influenced not just by geographical proximity but also by other socio-economic and logistical factors.

**Table 7:** The results of cooperation patterns.

Attractions	White Sand Lake-Xinjiang Museum	White Sand Lake-Flaming Mountain	Karez-Flaming Mountain	Nalati Grassland-Flaming Mountain
**Pearson Correlation Coefficient**	0.352	-0.233	-0.344	-0.346
**Pearson P-value**	0.218	0.422	0.229	0.226
**Granger Causality Coefficient (A→C)**	156.566	-	-	-
**Optimal Lag Days (A→C)**	3	-	-	-
**Granger P-value (A→C)**	0.000	-	-	-
**Granger Causality Coefficient (C2192A)**	4575.748	-	-	9.920
**Optimal Lag Days (C→A)**	2	-	-	2
**Granger P-value (C→A)**	0.0	-	-	0.009
**Cooperation Patterns**	Staggered Cross-Regional	No significant pattern	No significant pattern	Staggered Cross-Regional

A in parentheses represents Antecedents, C represents Consequents, → represents that A has a lifting effect on C in the association rules.

In summation, while tourist attractions within the core-intermediary and the intermediary-periphery exhibit significant cooperation patterns of staggered cross-regional collaborations, others, despite their geographical proximity and community affiliations, remain operationally autonomous. The results above demonstrate that inter-community, inter-level, and inter-region constitute the endogenous driving force of the tourism attraction potential cooperation network. These insights offer a nuanced understanding of the dynamics governing tourism demand, crucial for stakeholders aiming to optimize the tourist experience.

## Discussions

### General conclusions

Drawing on the digital footprints of tourists’ online engagement in travel decision-making and itinerary planning (Search index), this study has shed light on the existence of a potential cooperative network among tourist attractions within the destination ecosystem. Intriguingly, this network is delineated into three distinct communities, namely core, intermediary, and periphery, mirroring high, medium, and low tourist demand scales in that order. Such a configuration intimates a self-organizing potential cooperative network between tourist attractions, underpinned by congruencies in internal tourist demand and variances in external tourist experiences. From a functional standpoint, a directed continuum of cooperative prospects emerges among these communities. The core community, characterized by its pronounced tourist demand, ostensibly serves as a catalyst in amplifying the demand for other attractions. Occupying a central position in the network, the intermediary community not only forges ties with the core to augment its demand but also radiates its cooperative influence on the periphery. For the peripheral attractions, emblematic of the latent growth zones within the destination matrix, affiliating with the core and intermediary attractions appears to be a viable strategy for advancement. Delving deeper at the granular level, the evidence underscores a predominant trend of cross-regional and temporally offset collaborations among communities, accentuating the pivotal role of inter-community, inter-hierarchical, and inter-regional synergies in fueling the cooperative dynamics within the tourist attraction network.

### Contributions to knowledge

This paper proposes a methodology to mine potential cooperation networks among tourist attractions, an analytical tool is also provided for tourism stakeholders to gain insights into attractions’ roles, functions, and positioning. In contrast to the study of tourist attraction networks generated by the tourist movements between attractions [59], this paper is one of the few studies that provide new knowledge by uncovering the structure and internal potential cooperative interactions of network development from the origin side rather than the destination side [59, 60]. The emerging attractions potential cooperation network confirms that multilateral lifting relationships exist between attractions that extend beyond bilateral relationships. That inter-community, inter-hierarchical, and inter-regional cooperation patterns will significantly enhance connectivity between remote attractions and enlarge the scope of influence and the synergistic effect between destinations. It is an effort to expand the knowledge of tourist attractions potential cooperation network, addressing the call to apply big data to explore quantitative studies of tourist attractions potential cooperation network [61].

Furthermore, this study enhances existing research on tourist attractions potential cooperation network by integrating association rules learning and network analysis in terms of dynamics, systematicity, and mechanisms. Firstly, the framework considers the seasonal context in the tourism system and extracts peak periods from the temporal characteristics of destination tourism demand, thus eliminating noise from random tourism behavior and making the network more representative [62]. Secondly, within the attraction network, we identify a core-intermediary-periphery structure with significant differences, which exhibits positive feedback mechanisms that contribute to the potential cooperations within the destination through directed relational chains. The staggered cross-regional patterns between attractions provide micro-level explanations for the underlying cooperative mechanisms of the structure. The discovery of an intermediary between core and periphery and relationship patterns among attractions donate novel insights into the intrinsic mechanism of structure [63]. The tight, logical chain between attractions, communities, and networks advances the study by discussing hierarchical connectivity mechanisms within the network [64]. Furthermore, there is a close intrinsic logic between the tourism demand scale for tourist attraction and its function (connection) and roles (domain cooperation or receive cooperative impacts) in network. This study empirically demonstrates, through quantitative methods, that the collective travel decisions driven by the image perception of the destination from different source markets generate the potential cooperation network within the destination. Therefore, this framework is not only applicable to the China tourism system with Xinjiang as the destination, but also allows for extensive comparison and analysis of attraction networks in various countries and regions within the international tourism system.

Diverging from traditional survey data that captures travel behavior, we harness big data to discern the collective preferences of potential tourist groups across a more extensive region, thereby illuminating the intricate relationships and potential cooperative interaction mechanisms among tourist attractions. This innovative paradigm not only enhances sample coverage but also refines the temporal resolution of monitoring tourism intentions. Consequently, it facilitates a deeper exploration from the spatial relationships of attractions into their cross-regional teleconnections and interactions in time dimension, offering significant theoretical and practical implications for the coordinated development of tourism resources.

### Practical and managerial implications

In the context of large-scale tourism destinations, the implementation of a potential collaborative network among various tourism attractions holds substantial practical and managerial implications. First and foremost, the design of an efficient transportation network, informed by collaborative insights, becomes paramount. Such a network aims to ensure enhanced tourist mobility, alleviate congestion, and increase the overall density of the travel experience, thus improving the vitality of the tourism industry. Moreover, addressing the challenges posed by peak tourism periods becomes feasible through a well-coordinated network. This coordination allows for the strategic distribution of tourist traffic, mitigating the pressure on popular sites and transferring tourist demand to lesser-known attractions through the lifting effects uncovered in the network. Enhancing the tourist experience is another critical outcome. Potential collaboration seeks to foster the development of diverse and personalized tourism products across regions, significantly elevating visitor satisfaction. Concurrently, integrating shared market intelligence and customer data with the collaborative network paves the way for more effective marketing strategies, attracting a broader demographic. Balancing regional development emerges as a key benefit, ensuring equitable resource distribution and spotlighting underdeveloped areas. Furthermore, the network aims to enhance operational efficiency and synergy among different regions, sharing best practices and expertise. Lastly, fostering economic diversification within local communities is vital for the sustainable development of regional tourism. The network encourages innovative tourism products and services by monitoring dynamic tourism demand from the tourists’ side, reducing dependency on singular attractions and promoting a sustainable tourism ecosystem. In essence, these insights underscore the importance of integrated and sustainable development strategies in large-scale tourism management, highlighting the need for collaborative networks that not only enhance the visitor experience but also contribute to the socio-economic and environmental well-being of the destination.

### Limitations and future research

The following limitations need to be mentioned in this research. First, the emerging search engine brand index captures the brand index of attractions after August 1, 2021. In this paper, we acquired one year of daily data to analyze the study region’s tourism flows and attractions potential cooperation network. The limited study period may have caused us to mine inadequate cycle change features in the temporal dimension. Furthermore, in selecting attractions, the 3A-5A class attractions and landscaped driveway were selected as destinations. However, our research merely contained data on 50 percent of the attractions in the catalog, which was caused by the poor tourism demand for attractions not indexed by search engines. Thirdly, in the network construction process, we applied the Top 69 association rules for modeling, and the number of rules may have neglected a few disadvantaged tourist attractions. Consequently, a priori information to regulate the number of association rules is essential for creating a more extensive network for more accurate data mining.

Building on our initial findings, our forthcoming research endeavors will pivot towards integrating user-generated content into the intricate tapestry of cooperative networks among tourist attractions. By drawing a parallel between the network architectures of the tourist demand spectrum and its supply counterpart, we are poised to delve into the nuanced discrepancies bridging tourist inclinations (or underlying motivations) and the tangible tourist trajectories. Such a comparative lens will undoubtedly pave the way for a more robust evaluation and discourse on the viability of synergies among tourist attractions. Moreover, harnessing the prowess of cutting-edge artificial intelligence methodologies, notably graph neural networks, presents a compelling prospect for capturing the fluidic organizational interplays and the spatial symbiotic paradigms inherent within the tourist destination ecosystem. This innovative approach, we believe, will indubitably carve out a niche in the rapidly evolving domain of tourism networks. In addition, the analytical framework we proposed can be applied to other regions to give tourism administrations data-driven insights for efficient management and more flexible policies to adapt to variations in tourism demand.

## Supporting information

S1 File (DOCX)
