# Supplementary material for: Detecting potential cooperative network for tourist attractions in a destination using search data
Source: PLoS One. 2024 Feb 7;19(2):e0298035. doi: 10.1371/journal.pone.0298035 (PMC10849253; doi:10.1371/journal.pone.0298035)
Supplement: S1 File — (DOCX) [file pone.0298035.s001.docx]

**Supporting information**

The data that support the findings of this study are openly available in figshare at https://doi.org/10.6084/m9.figshare.24948225.
